# Pain management in zebrafish: Report from a FELASA Working Group

**DOI:** 10.1177/00236772231198733

**Published:** 2023-12-05

**Authors:** Lynne U Sneddon, Paul Schroeder, Ana Roque, Karin Finger-Baier, Angeleen Fleming, Simon Tinman, Bertrand Collet

**Affiliations:** 1 Department of Biological and Environmental Sciences, University of Gothenburg, Sweden; 2 Red Kite Veterinary Consultants, 30 Upper High Street, Thame, Oxon, OX9 3EZ, UK; 3 IRTA-SCR, C/al Poblenou, Spain; 4 Max Planck Institute of Neurobiology (now: Max Planck Institute for Biological Intelligence), Department Genes – Circuits – Behaviour, Martinsried, Germany; 5 Department of Physiology, Development and Neuroscience, University of Cambridge, UK; 6 The Mina & Everard Goodman Faculty of Life Sciences, Bar-Ilan University Ramat Gan, Israel; 7 Université Paris-Saclay, INRAE, 27048 UVSQ, VIM, France

**Keywords:** Anaesthesia, ethics and welfare, analgesia, laboratory animal welfare, pain measurement, fish, organisms and models

## Abstract

Empirical evidence suggests fishes meet the criteria for experiencing pain beyond a reasonable doubt and zebrafish are being increasingly used in studies of pain and nociception. Zebrafish are adopted across a wide range of experimental fields and their use is growing particularly in biomedical studies. Many laboratory procedures in zebrafish involve tissue damage and this may give rise to pain. Therefore, this FELASA Working Group reviewed the evidence for pain in zebrafish, the indicators used to assess pain and the impact of a range of drugs with pain-relieving properties. We report that there are several behavioural indicators that can be used to determine pain, including reduced activity, space use and distance travelled. Pain-relieving drugs prevent these responses, and we highlight the dose and administration route. To minimise or avoid pain, several refinements are suggested for common laboratory procedures. Finally, practical suggestions are made for the management and alleviation of pain in laboratory zebrafish, including recommendations for analgesia. Pain management is an important refinement in experimental animal use and so our report has the potential to improve zebrafish welfare during and after invasive procedures in laboratories across the globe.

## Gestion de la douleur chez le poisson zèbre: rapport d’un groupe de travail de la FELASA Résumé

Le poisson zèbre est de plus en plus utilisé dans les études sur la douleur et la nociception et les preuves empiriques suggèrent que les poissons répondent aux critères attestant, au-delà de tout doute raisonnable, qu’ils ressentent de la douleur. Les poissons zèbres sont adoptés dans un large éventail de domaines expérimentaux et leur utilisation est de plus en plus fréquente, en particulier dans les études biomédicales. De nombreuses procédures de laboratoire chez le poisson zèbre impliquent des lésions tissulaires susceptibles de provoquer des douleurs. Ce groupe de travail la FELASA a donc examiné les preuves de la douleur chez le poisson zèbre, les indicateurs utilisés pour évaluer la douleur et l’impact d’une gamme de médicaments ayant des propriétés analgésiques. Nous signalons qu’il existe plusieurs indicateurs comportementaux qui peuvent être utilisés pour déterminer la douleur, notamment la réduction de l’activité, l’utilisation de l’espace et la distance parcourue. Les médicaments analgésiques préviennent ces réponses, et nous mettons en évidence la dose et la voie d’administration. Plusieurs améliorations sont suggérées pour les procédures de laboratoire courantes afin de minimiser ou d’éviter la douleur. Enfin, des suggestions pratiques sont émises concernant la gestion et le soulagement de la douleur chez le poisson zèbre de laboratoire, notamment des recommandations pour d’analgésie. La gestion de la douleur est un perfectionnement important dans l’utilisation expérimentale des animaux et notre rapport a donc le potentiel d’améliorer le bien-être du poisson zèbre pendant et après les procédures invasives menées dans les laboratoires du monde entier.

## Schmerzbehandlung bei Zebrafischen – Bericht einer FELASA-Arbeitsgruppe Abstract

Es gibt empirische Belege dafür, dass Fische die Kriterien für Schmerzempfinden zweifelsfrei erfüllen, und Zebrafische werden zunehmend für Studien über Schmerzen und Nozizeption verwendet. Zebrafische werden in einer Vielzahl von Versuchsbereichen eingesetzt, insbesondere zunehmend in biomedizinischen Studien. Viele Laborverfahren mit Zebrafischen sind mit Gewebeschäden verbunden, die zu Schmerzen führen können. Daher hat diese FELASA-Arbeitsgruppe die Evidenz für Schmerzen bei Zebrafischen, die zur Bewertung von Schmerzen verwendeten Indikatoren und die Auswirkungen einer Reihe von Medikamenten mit schmerzlindernden Eigenschaften untersucht. Wir berichten, dass es mehrere Verhaltensindikatoren gibt, die zur Bestimmung von Schmerzen herangezogen werden können, darunter verringerte Aktivität, Raumnutzung und zurückgelegte Strecken. Schmerzlindernde Medikamente verhindern diese Reaktionen, und wir zeigen die Dosis und den Verabreichungsweg auf. Um Schmerzen zu minimieren oder zu vermeiden, werden verschiedene Verbesserungen gängiger Laborverfahren vorgeschlagen. Schließlich werden praktische Vorschläge für die Behandlung und Linderung von Schmerzen bei Zebrafischen im Labor gemacht, einschließlich Empfehlungen für Analgetika. Die Schmerzbehandlung ist ein wichtiger Faktor bei der Verwendung von Versuchstieren, und unser Bericht hat daher das Potenzial, das Wohlergehen von Zebrafischen während und nach invasiven Eingriffen in Labors weltweit zu verbessern.

## Tratamiento del dolor en el pez cebra: informe de un grupo de trabajo de la Federación de Asociaciones Europeas para las Ciencias del Animal de Laboratorio (FELASA) Resumen

Las pruebas empíricas sugieren que los peces cumplen los criterios para experimentar dolor más allá de toda duda razonable y el pez cebra se utiliza cada vez más en estudios sobre el dolor y la nocicepción. El pez cebra se adopta en una amplia gama de campos experimentales y su uso está creciendo especialmente en estudios biomédicos.

Muchos procedimientos de laboratorio en el pez cebra implican daño tisular y esto puede dar lugar a dolor. Por lo tanto, este grupo de trabajo de la FELASA revisó los indicios del dolor en el pez cebra, los indicadores utilizados para evaluar el dolor y el impacto de una serie de fármacos con propiedades analgésicas. Informamos que existen varios indicadores de comportamiento que pueden utilizarse para determinar el dolor, como la reducción de la actividad, el uso del espacio y la distancia recorrida.

Los fármacos analgésicos previenen estas respuestas, y destacamos la dosis y la vía de administración. Para minimizar o evitar el dolor, se sugieren varias mejoras en los procedimientos habituales de laboratorio. Por último, se hacen sugerencias prácticas para la gestión y el alivio del dolor en el pez cebra de laboratorio, incluidas recomendaciones para la analgesia.

El tratamiento del dolor es una mejora importante en el uso de animales de experimentación, por lo que nuestro informe tiene el potencial de mejorar el bienestar del pez cebra durante y después de los procedimientos invasivos en los laboratorios de todo el mundo.

## Background

### Definition of pain

The International Association for the Study of Pain has updated their definition of pain to ‘an aversive sensory and emotional experience typically caused by, or resembling that caused by, actual or potential tissue injury’.^1^ After reviewing the empirical published scientific evidence, many authors have concluded that fishes experience pain beyond a reasonable doubt (for example, Sneddon^2^). The acceptance of pain in fishes naturally has implications for their treatment. In the context of scientific experimentation, fish are protected under legislation in many countries, including the European Union, and researchers using fish models are legally obliged to avoid, minimise and alleviate pain where pain is not the objective of the study. However, there is a lack of data on analgesic use in fishes and there is a need for the development of pain management strategies, which is the subject of this report. Zebrafish have become a prominent model in a variety of experimental contexts and in some countries and regions account for half the numbers of fish used in experiments. (See ALURES database https://webgate.ec.europa.eu/envdataportal/content/alures/section1_number-of-animals.html; in 2019 over half a million zebrafish were used. but note that fin clipping and tagging is not reported in some countries; this decreased to over a quarter of a million in 2020 possibly due to the COVID-19 pandemic). From an evolutionary perspective and from the underlying molecular biology through to whole animal behaviour, pain and nociception is highly conserved from invertebrate groups through to fishes, birds and mammals.^3^ Zebrafish are now used as a valid model for the study of pain and so for this species to be a relevant proxy in biomedical studies they must experience pain.^4^ In invasive studies (where the study of nociception and pain is not the objective) this also means that the damage following these laboratory procedures may cause pain which could potentially present a confounding factor.^5^ This report fulfils a knowledge gap in producing a pain management guide for zebrafish in laboratory studies to allow researchers and carers to provide effective pain-relief as part of their experimental procedure.

Pain management protocols are relatively under-developed for fishes and notably for zebrafish, an important laboratory model.^6^ Anaesthesia is in use with recommendations on what to use and how to administer it.^7^ Analgesic drugs have been categorised as peripherally acting drugs (local anaesthetics), non-steroidal anti-inflammatory drugs (NSAIDs), and opioids and opiates, which have both a peripheral and a central effect.^4,6^ Analgesics are united by the fact that they offer symptomatic treatment rather than deal with the cause behind a painful event. This report will review the current state of the art on pain assessment, the procedures likely to result in pain, and ways of minimising and alleviating pain in zebrafish and provide protocols for implementation of pain management in the laboratory.

### Common laboratory procedures in zebrafish

Since zebrafish are adopted in a wide variety of experimental contexts they are subject to a wide variety of procedures which potentially cause tissue damage (summarised in Table 1^8,9^). Invasive procedures typically involve the use of anaesthesia to render the fish unconscious to allow the procedure to be conducted safely, efficiently and to safeguard the welfare of the fish. Typically, analgesia by administering drugs with pain-relieving properties is not adopted (for example, Sneddon,^5^ Chablais and Jaźwińska,^10^ Lemmens et al.,^11^ Schweitzer et al.^12^). Recent studies have provided compelling evidence that a range of tissue damaging procedures substantially alter zebrafish behaviour and that these responses are prevented by a range of analgesic drugs at effective doses.^13–20^ Severity of pain tends to be based on the duration, where pain which lasts a few hours is classed as acute and therefore mild; moderate pain lasts for many hours; and severe pain lasts for days, but intensity of pain can also be considered.^21^ Of course, pain could be intense for a short period then subside quickly, so duration informs us about the length of pain rather than its intensity. Pain can be gauged by the quantitative change in behaviour from normal behaviour (see Supplementary material online for discussion of the validity of pain assessment). Adult zebrafish typically reduce activity, swim less and spend more time at the bottom of their tank when experiencing a painful procedure.^17–20^ Thus, it is vital that we have a user-friendly and easy means of assessing the extent (intensity) and duration of pain so researchers and carers can intervene and provide pain-relief (analgesia) if this does not confound experimental data collection. Pain scales are available for mammals, such as the facial grimace scales, which can be employed to gauge intensity of pain. A pain scale does exist where fractal dimension of complex swimming trajectories of zebrafish reflects the exposure to increasing concentrations of a low pH chemical (acetic acid) where the complexity of zebrafish movement declines in a concentration dependent manner.^19^ The effect of passive integrated transponder (PIT) tagging and caudal fin clipping was also measured using this scale. Undisturbed controls (anaesthetised only) and fin clipped fish administered with analgesia had the most complex swimming behaviour compared with groups subjected only to procedures that involve pain. However, this analysis relies on video recordings and tracking software. Studies in other laboratories exploring genomic screening or amputation fin clips have confirmed these findings that caudal fin clipped zebrafish have an altered fractal dimension, spend more time in the bottom of the tank and decrease distance travelled and that immersion (where the drug is dissolved in the holding water) in lidocaine prevented these changes.^22–24^ Thus, we do have behavioural indicators for the assessment of pain and studies have now tested analgesic drugs and identified effective doses and administration routes (reviewed in Sloman et al.^6^). How we can employ these pragmatically to manage pain in laboratory zebrafish is discussed below. Non-pharmacological treatment in pain management of zebrafish remains an understudied area. Whether strategies such as environmental enrichment improve recovery of pain is unknown and future studies should address this important topic.

**Table 1.** Common laboratory procedures in zebrafish, their consequences and possible refinements. During and after these procedures, zebrafish should be monitored for signs of pain with analgesia provided where appropriate and practically possible.

Procedure	Consequences	Refinement
Handling (without net, e.g. with gloved hands)	Nociceptors in fish are excited by >0.1g (this is well below the mechanical threshold for humans and would reflect light touch), therefore handling may give rise to pain. Further stress is elicited due to removal from water and air emersion.	Keep handling to a minimum. If removed from water place fish on moist materials.
Netting	As above. Possibility of net abrasion exciting nociceptors.	Use of fine mesh knotless nets.
Fin clipping (above 20% of fin)	Fin clipping, where a portion of a fin is removed under anaesthesia, is a common method for genomic screening and for fin amputation studies or to generate and study painful stimuli.	Improved sequencing methods, where available, would allow a smaller portion of fin to be clipped. Alternatives: skin swabbing if suitable. Take a smaller portion of tail fin where possible under anaesthesia and where appropriate and practically possible the use of analgesia should be considered.
Fin clipping (below 20% of fin)	There are no empirical studies published on the consequences of fin clipping a smaller portion <20%. Therefore a precautionary approach should be applied	Improved DNA extraction and genotyping methods allow a smaller portion of fin to be biopsied and ideally less than 10% should be removed under anaesthesia. Where appropriate and practically possible the use of analgesia should be considered. Alternative: skin swabbing if suitable.
Tagging	Visual tags such as visible implant elastomer (VIE) can be injected under the skin to allow individual identification and may excite nociceptors. Passive integrated transponder (PIT) tags are available for zebrafish and can be inserted in the peritoneal cavity under anaesthesia and are known to result in pain-related behaviour. Tags can increase weight, drag and affect buoyancy, thus affecting swimming as well as causing local tissue injury and secondary infection.	Use of effective pain-relieving drugs after tagging is recommended. The use of 3.5–5 mg/l of lidocaine administered via immersion improved welfare after VIE tagging.^8^ Selection of size of PIT tag relative to animal size is critical. The smallest tag should be used in relation to the weight of the fish. If infection occurs veterinary treatment should be provided or euthanasia considered.
Surgical procedures	Minor surgery may include acute procedures such as fin clipping and tagging as described above. Major surgery usually refers to the penetration of the body cavity, or surgery that will cause significant physiological or physical disturbance to the fish. This is likely to excite nociceptors in the damaged areas.	Peri- and post-operative care is important in the success of a surgical procedure. Individuals should be carefully monitored during and after surgery, which may lead to euthanasia if adverse changes are seen. Analgesia should be adopted to minimise pain.
Toxicology	The fish acute median lethality test (LC50) may be used for testing the toxicity of novel compounds in some countries. EU legislation prescribes that death as the end-point of a procedure shall be avoided as far as possible and replaced by early and humane end-points. Where death as the end-point is unavoidable, the procedure shall be designed so as to: (a) result in the deaths of as few animals as possible; and (b) reduce the duration and intensity of suffering to the animal to the minimum possible. Chemicals such as acids with a low pH of <3 excite nociceptors on the skin of fish and may cause pain.	Identification of earlier humane end-points before mortality have been proposed.^9^ For zebrafish used in toxicology studies, this is aided by methodical measurements of behavioural and physiological welfare parameters. Use of analgesia may be advantageous if chemicals are acidic (pH <6). Any procedure that may result in suffering or mortality needs to be monitored frequently.
Infection studies	Studies that expose fish to pathogenic organisms may result in the health of the fish deteriorating and may be necrotic, causing tissue damage and possible pain.	Pain relief may be advantageous if tissue damage occurs as a result of the pathogen.
Imaging	Potential injury from positioning or from the heat of molten agarose which may excite nociceptors. Anaesthesia may not be suitable for electrophysiology or neuronal calcium imaging studies.	Careful capture and handling. Effective use of anaesthesia although the impact of repeat anaesthetic exposure (more than once) is not fully understood.
Genetic modification	The alteration of genetic material is prevalent in the zebrafish. Genetic modification may lead to deleterious and sometimes ultimately lethal phenotypes. It is possible some phenotypes may lead to pain.	Researchers should be aware of the potential phenotypes that may be detrimental to the welfare of the individual and may cause pain, e.g. osteoarthritic, cancer, physical deformity. Where appropriate and once a phenotype is known to be detrimental analgesia should be employed to alleviate pain.
Injection	Removal from water causing stress. Restraint and injection may cause pain where the fish struggles when anaesthesia is not used. Small needles should be used as zebrafish tissues could be damaged by needle sizes used for larger species.	Use of light sedation/anaesthesia to facilitate handling and rapid injection. If anaesthesia is not used pain relief should be considered.
Gamete (egg and sperm) collection	Handling and netting as above. Gamete collection requires gentle massage and pressure of anaesthetised adults to release gametes. May cause bruising or tissue damage.	Only skilled personnel perform the procedure. If pain is assessed in the animals after the procedure analgesia should be considered.

### Review of the methods for pain assessment in zebrafish

The definition of human pain stated above shows the difficulties associated with the evaluation of pain. Therefore, we adopt the definition of animal pain that sets out several criteria an animal must fulfil to be considered capable of experiencing pain.^25^ Pain cannot be generalised since it is not expressed in the same way by every individual and finally there is no gold standard to evaluate pain in animals. Therefore, pain can only be evaluated by integrating indirect parameters, which can be classified as general, physiological and behavioural indicators.

#### General (physical) indicators

These indicators refer to the physical appearance of the animal or to its biological functioning which can be altered as a result of pain. They are generally an indirect measurement since they result from sustained changes in physiological or behavioural parameters due to pain. General indicators contribute to the evaluation, but they cannot *per se* define/clarify whether the situation is painful. When they are related to biological function, they are probably more useful when they are unchanged (i.e. optimal functional indicators such as normal behaviour and demeanour, colour, no significant stress responses, healthy or disease free, good reproduction and growth performance), guaranteeing absence of pain.

#### Physiological indicators

These indicators refer to biochemical parameters or other clinical parameters measured in the individual animal which can be altered when the animal experiences pain. These indicators are related to two mechanisms: a) pain is a stressor which directly stimulates the release of hormones from the hypothalamic–pituitary–interrenal axis and the sympathetic system; b) tissue damage activates the immune system and the release of inflammatory mediators which may also activate the interrenal gland. Some of the substances released during inflammation (potentially a pain generating process) can be used as indirect indicators of pain.^26^ Physiological indicators have the same disadvantage as behavioural indicators; it is not easy to distinguish between a painful situation and stressful only situation unless the changes are more than a stress response. For example, opercular beat rate in rainbow trout and zebrafish after painful treatment is much higher than after stress.^27^ Generally, these variables can change rapidly over very short periods and may be influenced by the sampling itself. However, careful sampling should overcome this. For example, White et al. sampled cortisol from the tank water of zebrafish rather than disturb the fish to obtain a sample.^28^

#### Behavioural indicators

These indicators refer to deviations from the normal/established behaviour patterns of animals of the species and age under evaluation. Some lists of indicators which may indicate presence of pain in animals have been proposed.^27^ These lists are incomplete, however, and the parameters proposed are open to different interpretation by each observer if not properly trained.^3,27,29^ In addition, behavioural parameters are subject to variation between zebrafish populations and between strains including those genetically modified. However, recent studies on painful procedures in zebrafish have shown that after painful treatment the fish are much less active, with lengthy periods of immobility, use the bottom of the tank more and swim much less; these are easy behaviours to observe with the naked eye.^18–20,27^ They also must be verified to see whether they exist under non-painful conditions or under stressful only conditions so that they are indeed identified as specific to painful events since pain is inherently stressful.^20^ Performance of anomalous behaviours specific to pain have been observed in fishes, including zebrafish (see reviews^3,29^).

For the specific case of fish and even more particularly zebrafish, a large list of variables/parameters/indicators has been proposed to evaluate presence/existence of pain in fish as listed in Table 2.^17–20,27,28,30–34^

**Table 2.** List of parameters that have been used in zebrafish and other fishes to evaluate pain and stress.

Type	Indicator
General	Presence of lesions or wounds – these are assumed to be painful, therefore if present the animal should be in pain due to tissue damage.^30^
	Abrupt change of colour in skin and/or fins – stressed fish may blanch.^30^
	Increased mucus production.^30^
	Unresponsive to food.^30^
	Problems to maintain balance.^27,30^
	Altered use of space (time spent in the one-third top or bottom, % of the tank explored, % time near the walls) significantly different from basal levels.^17,18,31^
Behavioural	Altered levels of activity (total distance travelled, % time being active, % movements) significantly different from basal level.^18,32^
	Altered swimming speed (average speed, maximum speed, average acceleration, maximum acceleration. All these can be differentially recorded at top and/or bottom of tank).^18–20^
	Number of erratic movements (number of sharp, rapid, unexpected changes in direction during swimming/10 min and number of fish affected) significantly different from basal levels.^28,33^
	Lethargy, no response to stimuli.^30^
	Swim individually, a fish which is not swimming with the shoal.^20,30^
	No response to conspecifics.^30^
	Body curve index (hunched position).^31,32^
	Increase of tail beats that do not lead to propulsion in the water.^17,33,34^
	Number of freezing events (stop moving for more than 2 s).^31^
	Increased aggression from basal levels.^30^
Physiological	Increased cortisol levels.^28^
	Respiratory rate/operculum (gill cover) beat frequency.^27^

**Figure 1.**
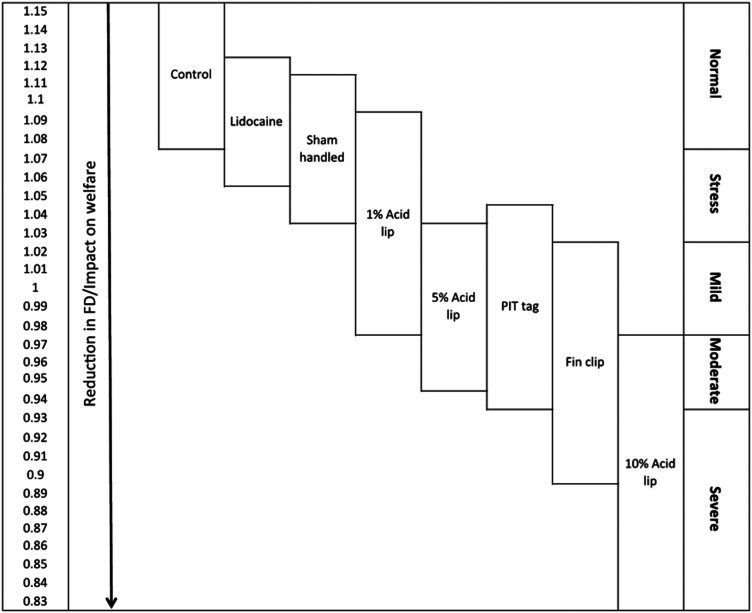
Pain scale in zebrafish. Hypothetical pain and welfare scale based upon Fractal Dimension (FD) analysis of complex swimming trajectories of zebrafish. Boxes indicate the range of FD values associated with each treatment group (control, sham handled (anesthetised and handled only), fin clip and lidocaine, fin clip, PIT tag, 1%, 5% and 10% acetic acid injected into the lip). A decrease in FD value indicates a reduction in welfare and an arbitrary scale of intensity represents zebrafish welfare as normal, stressed and in pain from mild to severe (reproduced under a Creative Commons Attribution (CC BY) licence from Deakin et al., 2019).^19^ PIT: passive integrated transponder

In order to develop a protocol to assess pain in a zebrafish, its reliability and validity must be estimated (see Supplementary material). None of the parameters listed (Table 2) before are, *per se*, sufficient to assess presence of pain and even less to estimate its intensity. For that, a system addressing complementary parameters must be employed, considering whether the fish are held individually, in pairs or in larger groups.^18–20,28^ Ideally, the combination of relevant parameters should cover the physical response to pain and behavioural parameters expressing discomfort. Here we restrict our report to juvenile (>5 days post fertilisation (dpf)) and adult zebrafish that fall under European and other regions’ legislation (see Supplementary material for brief guidance on younger forms) based on the evidence that larvae respond to a noxious challenge in a similar way to adult zebrafish.^13–16^ Deakin et al. assessed a limited number of painful procedures including tail fin clip, PIT tagging, subcutaneous injection of 1%, 5% and 10% acetic acid alongside undisturbed controls, sham handled animals (anaesthesia only) and fin clipped zebrafish administered with lidocaine as pain relief using a 3D tracking system in parallel with the Fish Behaviour Index, which can automatically provide a welfare status of Healthy, OK, Unhealthy and Abnormal.^18^ Many facilities may not be able to practically use these software tools and so assessment of pain must be done using a human observer. It may not be possible to recognise different categories of pain as that would require assessment of subtle changes in behaviour and in particular the magnitude of reduced swimming distance, activity and space use. In this case it may be possible only to recognise a change from normal behaviour after an invasive procedure and once any reduction in these parameters is observed then carers can administer pain-relief using the effective drugs and doses contained in this report. One study has produced a pain scale but again this was based upon fractal dimension analysis of the swimming trajectories of zebrafish before and after painful procedures so cannot be performed by a human observer at the tank side. This pain scale does provide us with some useful information, that the complexity of swimming is reduced according to the procedure (Figure 1).^19^ This pain scale could be developed in future studies exploring a wider range of procedures.

## Management of pain

### Analgesic drugs used in zebrafish

By January 2022, 183 papers had been published concerning the use of analgesics in fishes (Web of Knowledge; search terms analgesia and fish from 1990, 21/01/2022) with 93 of these pertaining to zebrafish. The three main classes of analgesic drugs (NSAIDs, opioids and local anaesthetics) have been studied as potential analgesic agents in larval^13–16^ and juvenile or adult zebrafish.^17–20^ These drugs provide pain-relief only and do not provide anaesthesia (sedation or unconsciousness) although local anaesthetics are used in doses far lower than those used for general anaesthesia. Other drugs are used in managing mammalian pain and are reviewed in the Supplementary material. Care should be taken that both temperature and pH of the water may affect the action of the drug; however, zebrafish are typically held at approximately 28°C and at a stable pH in a narrow range (7.0–7.4). The drug itself may affect the pH of the water and this should be corrected, for example by the use of a buffer.

### NSAIDs

NSAIDs are the most frequently used analgesics in human and veterinary medicine.^35^ They reduce pain by selectively or non-selectively inhibiting the two isoforms of cyclooxygenase, which converts arachidonic acid to a precursor of prostaglandin, thus targeting one of the three principal mediators of nociception. Perioperative administration of acetylsalicylic acid to zebrafish subjected to fin amputation resulted in a significant reduction of post-nociceptive behavioural patterns, compared with animals exposed to saline solution, although uptake of the molecule was not verified.^15^ For the same species, flunixin immersion modulated activity changes after fin clipping.^18^ Indomethacin injected intra-peritoneally significantly inhibited pain induced with formaldehyde injections into the tail^36^ and diclofenac prevented the behavioural responses and curved posture associated with abdominal pain.^37^

### Opioids

Opiates and opioids work centrally (as endogenous opiates such as endorphins) in the forebrain and medulla of mammals, dissociating the feeling of pain from a nociceptive stimulus. These compounds are full opioid agonists or classic opioids such as morphine, fentanyl, tramadol, levomethadone and oxycodone, which have a strong analgesic and sedative effect, and partial agonists providing pain relief only such as buprenorphine and butorphanol (agonist–antagonist).^38^ Opioid compounds produce analgesia by acting on opioid receptors (in the case of morphine primarily mu, but also delta and kappa – all of which have been verified in fish) located on spinal and cerebral neuronal cell membranes. Morphine is a potent mu-opioid receptor agonist which has shown to reduce pain-related behaviours after painful treatment in adult zebrafish at 48 mg/l, preventing behavioural responses to pain.^18^ Similarly in 5 dpf zebrafish larvae the same concentration of morphine dissolved in tank water prevented the decline in activity seen in response to painful treatment.^13–16^ Prior immersion of 5 dpf zebrafish larvae with 0.1 mg/l of the partial opioid buprenorphine before exposure to acetic acid prevented post-nociceptive behavioural changes. The same study also demonstrated that the antinociceptive properties of the drug buprenorphine could be reversed by concurrent treatment with the mu-receptor antagonist naloxone.^39^ Buprenorphine at 2.52 mg/l also completely inhibited both acute hot and cold temperature aversion and suppressed sensitised heat avoidance in 5 dpf zebrafish larvae.^40^

### Local anaesthetics

Local anaesthetics have been used in both human and veterinary medicine to relieve pain. They work by temporarily reducing the permeability of neurones for Na+ and K+ ions, interrupting the transduction of noxious stimuli.^41^ Due to the deactivation of ion channels required for depolarisation and repolarisation, the movement of action potentials normally triggered by the nociceptive event is inhibited and the transfer of noxious stimuli is interrupted. Lidocaine can be administered via injection into the site of damage. However, when used in conjunction with an immersion anaesthetic this may lead to mortality after surgery, possibly through systemic effects such as myocardial depression.^42^ This remains to be thoroughly tested in fishes. Lidocaine immersion at 2 mg/l and 5 mg/l reduced behavioural changes when used perioperatively on fin-clipped adult zebrafish; for this drug dose-dependent uptake was confirmed.^17^ Studies have confirmed the efficacy of lidocaine via immersion at 5 mg/l for individual^18,19,22–24^ and up to three group held adult zebrafish and for <5 dpf zebrafish^13–16^ but not for larger groups containing six individuals.^20^ Doses at 10 mg/l of lidocaine affected swimming behaviour in zebrafish so care should be applied to using concentrations that may cause unwanted side-effects.^43^

### Anaesthesia and/or analgesia in fish

Many of the procedures listed in Table 1 require anaesthesia, to immobilise the fish, ensure loss of consciousness and in some cases provide analgesia for imaging, manipulation or surgery. Since some anaesthetic agents also have analgesic properties, it is important to consider whether additional analgesia is necessary and whether the anaesthetic and analgesic drugs are compatible (see Supplementary material for a discussion of anaesthetic properties and monitoring of anaesthesia); for example, the use of benzocaine as an anaesthetic followed by the use of lidocaine to provide pain relief where both have the same mode of action (sodium channel blockers). It is possible that these two drugs could have an additive effect; however, this was an effective practice that reduced pain in zebrafish where behaviour was unaffected.^18–20^ The aim of general anaesthesia is to induce unconsciousness, analgesia and relaxation of skeletal muscles. In mammals, this is typically achieved by administering a combination of pharmacological agents targeting, amongst others, GABA and glutamate receptors and voltage-gated sodium channels.^42^ However, general anaesthesia in fish is often induced by the administration of a single agent and few studies have been performed to determine whether this is truly general anaesthesia or merely acting via neuromuscular block, causing paralysis without providing pain relief. Notably, one study has determined that tricaine (MS222) acts preferentially on neural but not muscle voltage-gated sodium channels in larval zebrafish.^44^ Further work is necessary to determine whether the same is true for other commonly used agents for fish anaesthesia and whether these drugs are centrally acting in zebrafish after blood–brain barrier formation. Discussion of suitable agents for induction of anaesthesia in zebrafish can be found in the Supplementary material. If the anaesthetic used does not provide analgesia, then use of an analgesic should always be considered in invasive procedures. However, anaesthetics have short lasting analgesic effects, so analgesia is recommended irrespective of the anaesthetic’s analgesic properties to ensure adequate pain relief is provided post-operatively. The analgesia could be administered via immersion before or during anaesthesia, but drug interactions should be considered. Another consideration is whether to fast (no food) the zebrafish prior to anaesthesia for 8–24 h (or overnight) to prevent regurgitation. If this period exceeds the overnight time period this may impair welfare by raising hunger levels so fasting should be kept to a minimum. This may be a practical decision since restricting food intake will reduce waste production (ammonia) during recovery, which may be particularly important if the animals are held in a closed or static tank.

The majority of studies have explored the impact of painful treatment for a few hours rather than days in zebrafish. One study followed fin amputation where the tail fin was clipped and found that the behavioural responses to this procedure, which included decreased distance travelled and increased time spent in the bottom of the tank, persisted for three days.^22^ Thus, pain assessment should be conducted at regular intervals after an invasive procedure and analgesia provided until the zebrafish shows recovery. Research investigating the duration of pain of different experimental treatments would be crucial to identify when zebrafish recover from each type of procedure and would assist in guiding more specific recommendations on for how long pain should be assessed and alleviated.

### Practical methods for the administration of analgesia in zebrafish

In zebrafish, the vast majority of anaesthetic and/or analgesic drugs are administered through immersion in system water, in which the active compound is dissolved (immersion) in the home tank or a separate tank or vessel. This approach can be considered as the most refined method or route of delivering analgesia since it does not involve handling the animal or invasive injections.^7^ Thus, the drug has a system wide effect on the animal. As not all compounds are soluble in water, pre-solving the compound in ethanol or dimethyl sulphoxide might be necessary, and the toxicity of these vehicles should be considered. Although the concentration of the drug solution can be exactly determined, it remains unclear what internal dosage will be reached through immersion. Another open question is whether immersion applied painkillers act through skin receptors (= locally) or centrally (by absorption through gills) and what effect this has on the analgesic properties of the compound. Immersion has been reported for all developmental stages of zebrafish.^4,6^ Immersion can present logistical issues since it may not be possible to anaesthetise in the home tank if the tank is part of a recirculating system or on a rack since this would expose other zebrafish to the drug. In this case it may be necessary to catch the fish and transfer it to a separate tank or vessel, which may be stressful. Alternatively, it may be possible to turn off the inflow of water into the home tank, add the analgesic drug and leave the flow off for a significant period of time for the analgesic to take effect. After this period the water would need to be sent to drain and replaced with system water at a rate to completely refill the tank before the tank water is recirculated. In commercial racking systems, tanks can easily be removed from the racking system. Studies using effective analgesia exposed the animals for 30–70 min prior to the procedure and the animals remained in the analgesic dose water for the duration of the 6 h experiment.^18–20^ However, behavioural changes seen in response to painful treatment were absent at 1 h after the procedure, suggesting that exposure to the drug for at least 90 min is necessary to prevent pain-related responses. In addition to immersion, for adult zebrafish intramuscular (i.m.), intraperitoneal (i.p.) and various intubation-based administration routes have been reported. Frequently, a prior anaesthetic immersion is used before the i.p. and i.m. techniques are applied. Intubation based administration seems to be the preferred choice for prolonged or repeated procedures, as it has a significantly lower mortality rate compared with extended or repetitive immersion event, for example see Martins et al.,^30^ and Wynd et al.,^45^ although Wynd et al. report a dynamic anaesthesia flow system for long-term imaging of adult zebrafish with improved survival rates.^45^ Analgesics or ‘painkillers’ can be administered prior to an invasive procedure, once the desired anaesthetic plane/stage has been reached or if the drug can be administered via immersion, then it is possible to administer prior to anaesthesia. Little has been reported in the literature regarding post-procedural care, unless post-procedural care is the topic of the study.^17,18^

### Proposed techniques to assess analgesic efficacy during perioperative care

When performing tissue damaging invasive procedures in fish, the likelihood of them experiencing pain and discomfort is probable based on empirical evidence in zebrafish^18–20^ and in other fish species.^2,3^ Thus, in these circumstances analgesic administration is advisable alongside anaesthesia. We suggest that perioperative care should adapt a multimodal approach combining anaesthetic and analgesic drugs. For zebrafish, due to their relatively small size, an immersion route is advisable to reduce manipulations, and a combination of drugs exhibiting different action times improves analgesia,^46^ although few studies have investigated combining anaesthesia with analgesic drugs (although see Valentim et al.,^47^ who used propofol combined with lidocaine at lower doses than if these drugs were used alone). If there is the slightest doubt that the fish is experiencing pain associated with tissue damage, then treatment with analgesics is recommended.^6^ However, the stress to the fish caused by chasing and netting to administer the analgesic might outweigh its benefit. Thus, administration during the procedure when the zebrafish is anaesthetised or administration via immersion in the experimental or home tank is preferable.

### Assessing analgesic efficacy

Typically, the responses to pain are used to assess the efficacy of analgesics since these should prevent pain-related changes in behaviour and physiology; for example, see Flecknell.^48^ If pain-relief is effective then no or only minimal signs of pain should be observed; however, if behavioural or physiological symptoms are apparent then the analgesic is either not effective or it requires a higher dose or re-dosing (Table 3^17,18,22,32,36,40,49^). Therefore, it is vitally important that we have a means of assessing pain in zebrafish and also empirical evidence to administer an effective dose of a pain-relieving drug. As mentioned previously, pain assessment is much better developed in mammals than in fishes. No recognisable pain scale has been recorded in fish but as described above there is a range of behavioural and physiological alterations that can be monitored. Of course, some of these indicators are also relevant for stress, anxiety and distress, thus judgement must be based on the premise that tissue damage has occurred.

**Table 3.** The three classes of analgesic drugs tested in adult zebrafish. The range of doses investigated, the species employed in the study, side effects including whether the analgesic prevented pain related changes and a comment on analgesic efficacy. Where not effective is stated these drugs did not prevent the changes in behaviour associated with painful treatment. The drugs were administered by intraperitoneally (i.p.), intramuscularly (i.m.) or via immersion in tank water. Injection volumes should be no more than 10 µl and immersion should be conducted for at least 70 min. Simplified tables are available in Supplementary material online for both adult and larval (<5 days post fertilisation) zebrafish.

Analgesic	Type of stimulus	Dose	Route	Prevents response to pain in measured variables	Side effects	Efficacy
Local anaesthetics						
Lidocaine	Tail fin clip/amputation^18,22^	1–5 mg/l	Immersion	Activity, space use and distance travelled	None observed	Immersion at 5 mg/l
Bupivacaine	Tail fin clip18	0.5–1.5 mg/l	Immersion		None observed	Not effective
Opioids						
Morphine	Formalin^36^	20 µl of 0.2 mg/ml	i.p.	Locomotion	None observed	Effective
	Fin clip^18^	3 mg/l and 48 mg/l	Immersion	Activity, space use and distance travelled	None observed	Immersion at 48 mg/l
	Acetic acid^32^	1.25–2.5 mg/kg	i.m.	Distance travelled	None observed	2.5 mg/kg
	NaCl applied to the cornea^49^	1–16 mg /kg	i.p.	Activity	None observed	16 mg/kg
Buprenorphine	Heat^40^	0.01–0.3 mg/kg	Immersion	Time spent in hot zone	None observed	Immersion at 0.3 mg/ml
NSAIDs						
Aspirin	Tail fin clip/amputation^17^	1–2.5 mg/l	Immersion	Activity	None observed	Immersion at 2.5 mg/l
Diclofenac	Abdominal pain^32^	40 mg/kg	i.p.	Body curvature, entry and time spent at the top of the tank, distance travelled and freezing duration	None observed	40 mg/kg
Flunixin	Tail fin clip/amputation^18^	2–8 mg/l	i.p.; immersion	Activity, space use and distance travelled	None observed	8 mg/l effective in adults only
Indomethacin	Formalin^36^	20 µl of 0.2 mg/ml	i.p.	Activity	None observed	Effective

NSAID: non-steroidal anti-inflammatory drug; i.m.: intramuscular; i.p.: intraperitoneal

Deakin at al. investigated a range of analgesic drugs and identified those that were effective in individual fish via immersion (lidocaine 5 mg/l, flunixin 8 mg/l and morphine 48 mg/l; note that Schroeder and Sneddon^17^ also tested 2.5 mg/l of aspirin).^18^ These drugs and doses prevented the pain related changes in behaviour with zebrafish displaying normal activity, swimming behaviour and tank space use. For fin clipping in group held zebrafish, lidocaine 5 mg/l was effective in up to three within a group of six individuals but not when all six in the group were clipped.^20^ This may have been due to uptake rates and the dose of 5 mg/l of lidocaine not constituting a high enough dose. Again, group held fish showed reduced activity and increased use of the bottom of the tank. Therefore, changes in activity and tank space use can be easily captured through direct observation to assess whether pain is occurring. At the same time administration of an effective analgesic drug via immersion would be minimally invasive to the fish. Carers and researchers may require training to identify the baseline behaviours of zebrafish so that they can reliably recognise post-procedural changes. If immersion is practically impossible then other routes such as i.m. and i.p. may be advisable (see Table 3) that could be administered during the procedure. Very little pharmacological information exists in fishes but exposure to lidocaine via immersion was detected 90 min after administration in fin clipped zebrafish.^17^ If still present in the tissues at 90 min lidocaine may still be active and continuing to provide pain-relief; however, this has yet to be investigated. Monitoring of pain and re-administration of analgesia should be based upon real time assessment at the tank side, that is, after first administration the drug should be readministered when the signs of pain reappear. Future studies should explore pharmacological properties of analgesic drugs and in particular the half-life of each drug at a specific dose to inform when the drug could be readministered. Side effects are also an important issue that may confound experiments. In the studies cited above no behavioural side effects were observed in zebrafish but there is a paucity of data on physiological, cellular or molecular responses. Scientists may need to explore whether the drugs employed affect data collection and whether zebrafish show avoidance to these drugs in the water. Researchers need to test the impact of pain and pain-relieving drugs in each different type of procedure or experiment to identify which drug is most effective and at what dose, and to ensure that the drug itself does not affect the outcome of the experiments.

### Protocols leading to good pain management

In all invasive procedures performed in zebrafish, researchers should adopt pain assessments during and after procedures and consider the administration of analgesia where pain responses are observed. The good management guide below is not an exhaustive list but seeks to provide recommendations for the most commonly performed protocols in zebrafish. This guide covers protocols to reduce both painful and stressful events (see Table 1). Assessment of pain could be done by employing score sheets where general, behavioural and physiological observations can be recorded (see Martins et al. for an example of a score sheet^46^). Unfortunately, there is very little research on analgesia in zebrafish so many questions remain unanswered. For example, should drugs be provided once, intermittently, or continuously? Further, there is little pharmacological information on the half-life of these drugs or persistence in the tissues to guide decisions about when to readminister analgesia. We hope this report will stimulate research in these important areas.

#### General handling

Handling should generally be kept to the absolute minimum.

1. Whenever fish need to be transferred between receptacles (e.g. tanks or containers), fish should be transferred by carefully and slowly pouring water between containers or tanks. Transfer time should be kept as short as possible to reduce any stress.
2. If sorting of individual fish is required, use knotless fine mesh nets in order to avoid exciting skin nociceptors by net abrasion. Gently and calmly catch the desired fish and keep air exposure to a minimum to reduce escape behaviour, which can lead to increased net abrasion. Consider the use of vessels that retain water to avoid air emersion if practically possible.
3. Only trained personal should handle the fish to minimise stress and subsequently escape behaviour. Clean gloves should be worn, and the fish kept moist by the use of wet paper towel or wet sponge.
4. When pipetting larvae and young juveniles, suitable pipettes should be used. Pipettes should have an appropriate diameter, so larvae can be pipetted without touching the pipette wall. Pipettes also should have a smooth rim to avoid skin injuries upon accidentally touching the fish while pipetting.

#### Genomic screening

Researchers should consider alternatives such as skin swabbing or use of genotyping at embryonic larval stages to replace fin clipping for obtaining DNA samples. If fin clipping is determined to be the only suitable technique, researchers should refine techniques to remove the smallest amount of tissue (<20% of the caudal fin but ideally <10%) under anaesthesia and analgesia should be provided if researchers identify pain indicators (Table 2) resulting from their fin clipping technique.

1. Skin swabbing has been validated as a non-invasive alternative for the collection of DNA samples in adult AB strain zebrafish and other species.^23,24,50^ When samples of skin mucus are collected, these contain sufficient sloughed off epithelial cells to extract gDNA. Care should be employed when handling fish since pressure above 0.1 g may be applied in swabbing and this pressure excites nociceptors. To maintain integrity of the mucus barrier and prevent dislodgement of scales, only a light swab should be applied by properly trained personnel. Tilley et al. demonstrated that skin swabbing triggers fewer changes in stress axis activation, behaviour and gene expression compared with fin clipping.^23^ However, some researchers report inadequate DNA collection using this method. Anecdotal evidence from our own laboratories and from discussions with colleagues suggest that this method may not be ideal for young zebrafish (<3 months) and it is advisable to use anaesthesia for easy handling of the fish if the zebrafish strain is particularly active. Pain relief may also be a consideration after skin swabbing has taken place. Only one study has investigated the impact of lidocaine on AB strain adult zebrafish and found that behaviour was similar to control, undisturbed fish and there was no difference between skin swabbed fish with and without lidocaine.^24^
2. Several methods have been published using gentle agitation or enzyme exposure to remove cells from living embryos or larvae thereby allowing DNA collection and genotyping non-regulated stages.^51–53^ These approaches allow for large numbers of embryos/larvae to be genotyped and only those of the correct genotype to be grown up, thereby reducing the number of animals reared, subjected to a procedure (for DNA collection) and then culled if they are not of the correct genotype. Larvae exposed to these procedures display normal behaviour and viability.^51,53^ However, further validation may be required for certain aspects of biology to ensure that these procedures do not result in lasting changes that would affect experimental outcomes.
3. If fin clipping is considered the only suitable technique, the biopsy should be no more that 20% of the caudal fin and ideally less than 10%. This procedure should be conducted under anaesthesia and where appropriate and practically possible the use of analgesia should be considered. This refinement has been adopted by many of the larger zebrafish facilities and it is important that this good practice is more widely adopted. Audira et al. explored tail fin clip as a model for amputation (>20% removal) and confirmed previous studies that this does significantly affect behaviour and that the alterations were seen over three days, thus researchers may need to consider longer-term pain-relieving protocols.^22^ There may be a misconception that taking smaller biopsies requires more sophisticated DNA extraction methods. The extraction method developed by Meeker et al. and used by the Sanger Centre to identify mutants in the Zebrafish Mutation Project is inexpensive and reliable and requires only sodium hydroxide, EDTA and Tris-HCl.^54,55^ This DNA extraction method works well for all genotyping reactions, yields a large amount of gDNA, so is therefore suitable for running multiple reactions, and has been demonstrated to be effective with even extremely small tail tip biopsies from larvae as young as 3 dpf.^56–58^

#### Surgical procedures

Surgical procedures are likely to result in pain and/or lasting harm. Such procedures may include (but are not limited to) creating lesions, removal of tissue, wounding, enucleation and tumour induction (reviewed in Gemberling et al.^59^).

1. The researcher should include pain assessments in their protocols (see Table 2).
2. If assessments indicate that the procedure is painful, protocols should be refined to include analgesia, with the appropriate assessments of their efficacy (see section on peri-operative care above).

#### Infection studies

Protocols that expose fish to pathogenic organisms by immersion or injection and where the deterioration of the fish health is an experimental measure will likely result in pain, suffering distress or lasting harm.

1. The researcher should include pain assessments in their protocols (see Table 2).
2. If assessments indicate that the procedure is painful, protocols should be refined to include analgesia, with the appropriate assessments of their efficacy (see section on peri-operative care above).
3. Validation studies may be necessary to ensure that the analgesic agent does not affect experimental parameters (such as the response of inflammatory cells) and may require the selection of analgesics that block nociceptors but do not have anti-inflammatory effects (see Supplementary material for a range of other drugs with analgesic properties).

#### Oral gavage

This method administers precise quantities of a specific concentration of a substance into the gut and is conducted under anaesthesia. If not performed correctly this could lead to damage and potential pain as well as mortality.

1. Researchers must be properly trained in the gavage method (see Collymore et al.^60^ for a video demonstration on adults and Cocchiaro and Rawls^61^ for gavage in 6–7 dpf zebrafish).
2. Careful netting, handling and anaesthesia as previously described.
3. Fish should be kept moist during handling and gavage. Adults can be placed into an incision in a wet sponge and larvae may need to be embedded in agarose (see section Embedding live fish below).
4. Recovery should be monitored prior to returning the fish to the home tank.
5. Pain assessment should be conducted after gavage to determine whether pain relief is required.

#### Embedding live fish

1. While orienting the fish, do not touch the fish itself, move it by gently moving the embedding solution around the fish.
2. If embedding in agarose, use only low melting point agarose. Let agarose cool down as much as possible before embedding fish. Higher temperatures can trigger heat-sensitive nociceptors.^40,62^
3. Sedation may be used if fish struggle during embedding, for example, because of age or genetic background. Struggling may increase the risk of accidental injury. Aversiveness of the sedative has to be balanced against the benefit of sedation and possible influence on experimental outcome. In imaging experiments, the protocol may require that sedation has worn off and the anaesthetic is no longer active. This may require several hours before the experiment can commence so the results are not confounded by the anaesthesia. Fish should be carefully monitored, and optimal environmental conditions maintained during this period.
4. Older fish may suffer from hypoxia while being embedded. Measures need to be taken to avoid hypoxia, for example, provide additional oxygenated water via intubation, freeing the gills to allow respiratory movements or bathing the embedded fish in (super) saturated oxygenated fish water (see Kappel et al.^63^ for the methodology).
5. Freeing the tail by removing agarose allows embedded fish to show escape behaviour, which can aid the researcher in detecting aversive behaviour caused by distress or pain.

#### Gamete (egg and sperm) collection

1. Procedure should only be performed by trained and skilled personnel. Published methods suggest two or three people are required for collecting and handling samples for cryopreservation.^64^
2. Careful netting, handling and anaesthesia as previously described.
3. Healthy anaesthetised adults will release gametes upon gentle pressure. If no gametes are produced, adults should be moved immediately to a recovery tank. Applying increased pressure will not result in viable gamete production but will cause tissue damage to the adult.
4. During recovery from anaesthesia, researcher should include pain assessments in their protocols (see Table 2). Any animal showing pain, distress or suffering should be culled immediately by following an approved, humane method.
5. If assessments indicate that the procedure is painful, protocols should be refined to include analgesia, with the appropriate assessments of their efficacy (see section on peri-operative care above).

## Conclusion

Many experimental procedures may cause damage to laboratory zebrafish and it is our consensus that pain should be monitored, assessed and, when pain occurs, pain relief via analgesia should be provided where practically possible and where it does not confound the aims of the study. Researchers should adopt a cautionary approach and use both anaesthesia and analgesia protocols, especially in procedures where tissue damage is likely. Adopting pain management protocols in the use of laboratory zebrafish presents a major refinement in the use of a large number of animals under the 3Rs ethical principles.

## Supplemental Material

sj-pdf-1-lan-10.1177_00236772231198733 - Supplemental material for Pain management in zebrafish Supplemental material, sj-pdf-1-lan-10.1177_00236772231198733 for Pain management in zebrafish by Lynne U Sneddon, Paul Schroeder, Ana Roque, Karin Finger-Baier, Angeleen Fleming, Simon Tinman and Bertrand Collet in Laboratory Animals

## Data sharing statement

This report did not collect any data and is a report of a working group
