# Supplementary material for: Pain management in zebrafish: Report from a FELASA Working Group
Source: Lab Anim. 2023 Dec 5;58(3):261–76. doi: 10.1177/00236772231198733 (PMC11264547; doi:10.1177/00236772231198733)
Supplement: sj-pdf-1-lan-10.1177_00236772231198733 - Supplemental material for Pain management in zebrafish [file sj-pdf-1-lan-10.1177_00236772231198733.pdf]

Supplementary Information

Definitions: Validity and Reliability of Pain Assessment

Reliability: A reliable assessment tool is one that yields similar results when applied to the same individual experiencing the same level of pain at different times (test-retest reliability or intra-observer reliability) and when applied by different raters (inter-rater or inter-observer reliability). Reliability measures the ratio of true variance in the test to total variance. In a perfect test, true and total variance are the same, and the ratio will be 1.[1]

Validity: Validity assesses a tool's capacity to quantify the construct it is designed to measure. Validity can be divided broadly into three concepts:

- face and content validity – a judgement (not assessed empirically) that the tool is assessing what it purports to; assessed subjectively
- criterion-related/concurrent validity – correlation of results of the tool compared with an existing gold standard test; without a gold standard, this form of validity cannot be established
- construct validity – a tool's capacity to objectively assess the construct that it sets out to assess. This can be established by determining whether the tool can distinguish between a group known to have pain and one that does not. It can also be established by examining whether the tool yields results that vary appropriately according to changes in pain intensity, for example as a result of treatment.[2]

Pain Assessment in Larval Zebrafish

Larval (typically less than 5 days post fertilisation (dpf) but see below) have been used in the study of nociception and pain. The types of parameters assessed are listed:

- Reduction of activity 0-6 dpf (number of movements)[3,4]
- Reduction in speed of swimming (5 dpf, mm/sec)[4]
- Increased distance travelled, 5 dpf (measured as cm or mm/10sec)[5,6], effect reduced after use of medicine (4 dpf)[5]

- Reduction of time being active (5 dpf, %), seems to be a more sensitive parameter, this alteration was prevented with the use of analgesics[4]
- Altered swimming speed (5dpf, up or down depending on stimulus)[7]
- Increased thigmotaxis (5 dpf)[8,9]
- Increased heart rate (5 and 7 dpf, not 4 dpf, normoxia at 25ºC =180 beats/min 2-14 dpf)[10,11]
- Ventilation rate is altered but the noxious stimulus was lack of oxygen or hypoxia (3-5 dpf)[12]

A range of drugs have been tested within the above cited studies in larval zebrafish to determine if these reduce behavioural responses to potentially painful treatment and these are listed in a simple summary below (Table 1).

Table1. Effective pain-relieving drugs used in larval zebrafish (<5 dpf) administered via immersion.

Drug Class	Drug	Effective Dose	Reference
Local anaesthetic	Lidocaine	5 mg/L	Lopez-Luna et al. 2017[4,7,8]
Opioid	Morphine	48 mg/L	Lopez-Luna et al. 2017[4,7,8]
	Buprenorphine	5 µM<br>0.1 mg/L	Steenbergen & Bardine 2014[6]<br>Curtright et al. 2015[13]
NSAIDs	Acetylsalicylic acid	2.5 mg/L	Lopez-Luna et al. 2017[4,7,8]

Drugs with analgesic properties

Table 2 provides a simple summary of drugs with pain-relieving properties for adult zebrafish – see the main text for references. For injection a maximum of 10 µl is proposed and for immersion where the drug is dissolved in the holding water. Deakin et al. immersed for 70 minutes and demonstrated that the effective drugs and doses prevented behavioural changes in response to painful treatment.[14] Below we highlight a range of drugs with analgesic properties that were demonstrated in zebrafish or other fishes.

Table 2. Effective pain relieving drugs used in adult zebrafish administer via immersion, injection intraperitoneally (IP) or injected intramuscularly (IM). For references, please see Table 3 of the main publication.

Drug Class	Drug	Effective Dose
Local anaesthetic	Lidocaine	5 mg/ L
Opioid	Morphine	48 mg/L<br>8 mg/kg IP<br>5 mg/kg IP<br>2.5 – 5 mg/kg IM
NSAIDs	Acetylsalicylic acid	2.5 mg/L
	Diclofenac	40 mg/kg IP
	Flunixin	8 mg/L
	Indomethacin	5-20 µl IP with 200 mg/L

The following drugs are used in mammals to provide analgesia and it is possible with further testing they could be administered to zebrafish.

Medetomidine

The (alpha-2 adrenoceptor agonist) medetomidine is used as an anaesthetic pre-med with strong sedative and analgesic properties Medetomidine injected intramuscularly (IM) at 0.025 mg/kg into goldfish reduced the amount of additional anaesthetic needed and thus demonstrated an anaesthetic-sparing effect where lower concentrations of MS222 could be used in combination with medetomidine.[15] However, rainbow trout exposed to 6mg/l medetomidine became sedated rather than anesthetised.[16] Medetomidine combined with ketamine (100 µg/mL ketamine + 1.25 µg/mL medetomidine) induced a rapid surgical anaesthesia in zebrafish but recovery was more prolonged than MS222.[17] Further studies are required to understand the analgesic properties of medetomidine and its use in combination with other drugs.

Clonidine

In an assay exploring the use of small molecules with known analgesic properties as fish analgesics, 5 dpf zebrafish larvae sensitised with mustard oil were subjected to Clonidine immersion (5µM) and displayed an inhibition of acute and sensitized temperature aversion.[13]

Amitriptyline

Amitriptyline is a tricyclic antidepressant that is commonly used to treat chronic pain in humans.[17] Immersion with this drug (0.5μM, dissolved in DMSO), successfully mitigated thermal aversion in 5 dpf zebrafish.[13]

Feprosidnine

Feprosidnine is a stimulant drug which was developed in the Soviet Union in the 1970s; injected intraperitoneally in cod at 15-100 mg/kg this compound showed a substantial and strongly dose-dependent reduction in nocifensive behaviour after challenge with electric shocks.[19]

Anaesthesia in zebrafish

The following are recommended as suitable agents for inducing general anaesthesia in fish and some drugs have analgesic properties. See Table 3 for anaesthetics not described in detail and Table 4 for anaesthesia indicators.

Tricaine/MS222 and benzocaine

Tricaine (MS222) and benzocaine are members of the amine family of voltage-gated sodium channel blockers and, in other species, are more commonly used as local anaesthetic agents.[20] Tricaine is easily soluble in water or embryo medium, but the resulting aqueous solution has low pH and therefore requires buffering with the same amount of sodium bicarbonate.[21] Both agents are avoided by zebrafish and medaka in behavioural assays.[22-24] However, since tricaine has a history of safe use in zebrafish, and that its efficacy, safety and recovery profile is superior to the other commonly used anaesthetic agents, it remains the preferred anaesthetic agents for most researchers and in some countries, is one of the only approved anaesthetics for use in fish.[25] Researchers must check the regulations and availability of drugs in their own country since this can vary widely. Since tricaine and benzocaine are used at sufficiently high concentration to induce general anaesthesia, there would seem to be little pharmacological rationale for using drugs of the same class (e.g. lidocaine and novocaine) for peri-operative analgesia. Indeed, there is the possibility that the administration of such agents of the same pharmacological class may be more likely to result in over-dosing if used in combination, but this has been done for zebrafish where benzocaine was used as anaesthetic and a low dose of lidocaine (5 mg/L) provided effective pain-relief.[14,26] However, there is a case for using combinations of agents, where the drugs in question act via different receptors. Such an approach is commonly used in mammals (see main text). One such study in zebrafish demonstrated propofol/lidocaine combined use induced a quicker loss of equilibrium, and loss of response to light and painful stimuli compared with

MS222.[27] Since propofol acts via GABA receptors whereas lidocaine works via voltage-gated sodium channels, it is possible that these drugs could be used at lower concentrations than when used singly. It is also necessary to consider the half-life of anaesthetic drugs with analgesic properties since it is possible that the analgesic effects may persist after recovery from anaesthesia if the agent used has a long half-life. Studies on the pharmacokinetics of these drugs are limited in fish. Further environmental (e.g. temperature, salinity) and biological factors (e.g. developmental stage, body condition) will affect both the action and half-life of anaesthetics (review[28]). Although there is little information about half-life, adult zebrafish recover equilibrium within ~90-140 seconds after anaesthesia with tricaine, suggesting that it may be rapidly metabolised or excreted.[25,29] Although pain reflexes are abolished during the period of anaesthesia, this rapid recovery indicates that additional peri- and post-operative analgesia should be considered.[25] The number of times a fish is anaesthetised during one experiment is an important factor to consider in case repetitive anaesthesia and handling impairs the welfare of the fish. However, this may be beneficial if it results in a reduction in the number of animals used.[30] Caution should be applied since it has been reported that daily MS222 exposure can cause mortality.[31]

Other anaesthetic agents, such as clove oil (eugenol and isoeugenol), etomidate and metomidate, quinaldine sulphate, ketamine, alphaxalone, have been reported as suitable for use in fish. For many of these agents there is very little data on their efficacy or safety in zebrafish and are therefore less relevant for consideration here but are included in the table below as potential alternatives.

Table 3. Overview of other agents used as anaesthetics for fishes, with notes on their use, aversiveness and reference. Drugs in **bold** have been used in zebrafish.

**Drug**	**Reported use**	**Aversive**	**Additional notes**	**References**
**2-Phenoxyethanol (2-PE)**	Ornamental / hobbyist sedative, anaesthetic and euthanising agent	Moderately aversive (less than HCl)	Available in Europe as Aqua-Sed ®	Schroeder et al., 2021[32]<br><br>Readman et al., 2013[22]<br><br>Owen & Welsh 2021[33]

Quinaldine sulphate	Suitable for use in tropical marine fish and in transport of fish	Strongly aversive (more than HCl)	No analgesic properties	Stoskopf & Posner, 2015[34]<br><br>Readman et al., 2013[22]
Ketamine	NMDA receptor antagonist	Not tested	Available for research purposes in UK and other European countries but restricted access in some countries or not available in some territories<br><br>(e.g. UK Misuse of Drugs Act;USDEA Schedule III)	Ganjoor et al. 2021[35]<br><br>Bruecker & Graham 1993[36]
Ketamine – Xylazine Combination	Ketamine: NMDA receptor antagonist<br><br>Xylazine: analogue of clonidine and an agonist at the α_2 class of adrenergic receptor	Not tested	As above for ketamine. Longer induction times compared with each drug used on its own but longer duration of anaesthesia	Al-Hamdani et al. 2010[37]

**Clove oil (Eugenol and derivatives)**	Unknown mechanism of action (recommended in UK by RSPCA for humane killing of pet fish – largely because it is available from pharmacy and health stores without restriction, so easy to obtain).	Not aversive in bass and bream and fish recover well.<br><br>Evidence for being aversive in zebrafish (equal to MS222 in one study; less than MS222 in another)	Highly popular in non-zebrafish research centres.<br><br>Aqui-S® (Isoeugenol & polysorbate) is approved for use in fish farms in Australia, Chile, New Zealand, Norway and Vietnam.<br><br>Human health and safety concerns since Methyleugenol is carcinogenic.	Mylonas et al., 2005[38]<br><br>Readman et al., 2013[22]<br><br>Wong et al., 2014[24]<br><br>Musk et al. 2020[39]
**Alfaxalone**	Alfaxalone is chemically alfaxalone-2-hydroxypropyl-beta-cyclodextrin (HPCD). It is a synthetic neuroactive steroid that interacts with gamma-aminobutyric acid (GABA) receptors in the CNS to produce anesthesia and muscle	Not tested	Anaesthesia reported in Koi carp.<br><br>Reported as suitable for anaesthesia in zebrafish by immersion.	Minter et al., 2014[40]<br><br>Farry et al., 2022[41]

	relaxation.			
**Metomidate / Etomidate**	Imidazole-based non-barbiturate hypnotic drugs which act on activation and modulation of the inhibitory gamma-aminobutyric acid type A (GABAA) receptors.	Medaka and rainbow trout show no avoidance to metomidate. Common carp do show avoidance.<br><br>Zebrafish show no avoidance in one study[22] but in another study zebrafish avoided etomidate[43]	As hypnotics they have no analgesic properties and do not provide surgical level anaesthesia thus their use should be limited to non-invasive procedures.<br><br>Suppression of cortisol release means they present a confounding factor for studies investigating physiological stress.	Review in Martins et al. 2018[42]<br><br>Martins et al.

			Respiratory depression was observed in zebrafish	2018[17]

Table 4. Descriptions of the stages of anaesthesia and the parameters used to monitor anaesthesia in fish. A number of procedures are provided as examples of what can be done to the fish under these anaesthetic stages (*some authors suggest there is an intermediate stage between light and surgical termed medium plane anaesthesia; **an example of a reflex response is the fish swimming in response to a tail pinch; ***usually accompanied by the use of artificial ventilation where the gills are irrigated with fresh or anaesthetic dosed water (Reproduced with kind permission from Elsevier from Sneddon 2012).[28]

STAGE	PLANE	LEVEL OF ANAESTHESIA	GENERAL DEMEANOUR	ACTIVITY	EQUILIBRIUM	GILL VENTILATION RATE	REACTIVITY	HEART RATE	EXAMPLES OF PROCEDURES
0		Normal	Normal	Normal	Normal	Normal	Normal	Normal	
I		Lightly sedated	Disorientated	Reduced	Normal	Normal	Reduced	Normal	
II		Excitation	Agitated	Increas	Difficulty	Increased	Increased	Increased	

				ed					
III	1	Light anaesthesia	Anaesthetised	None	Loss	Decreased	Reflex responses**	Normal	Weight; close visu inspection; external invasive tags, skin or scrape
	2	Surgical*	Anaesthetised	None	Loss	Shallow	None	Reduced	Invasive tags; tissue re injection; blood sam gill biopsy, lesion dre recovery surgery*
	3	Deep	Anaesthetised	None	Loss	Rare movements	None	Reduced	Non-recovery surge
IV		Overdose	Apparently dead	None	Loss	None	None	Cardiac failure	

References

1. Jerosch-Herold, C. Assessment of Sensibility after Nerve Injury and Repair: A Systematic Review of Evidence for Validity, Reliability and Responsiveness of Tests. Journal of Hand Surgery 2005; 30: 252–264.
2. Jensen MP. The Validity and Reliability of Pain Measures in Adults With Cancer. J Pain 2003; 4: 2-21.
3. Huang, IJ, Sirotkin, HI, McElroy, AE. Varying the exposure period and duration of neuroactive pharmaceuticals and their metabolites modulates effects on the visual motor response in zebrafish (*Danio rerio*) larvae. Neurotoxicol Teratol 2019; 72: 39-48.
4. Lopez-Luna, J, Al-Jubouri, Q, Al-Nuaimy, W, Sneddon LU. Impact of analgesic drugs on the behavioural responses of larval zebrafish to potentially noxious temperatures Appl Anim Behav Sci 2017; 188: 97–105
5. Ko, MJ, Ganzen, LC, Coskun, E, et al. A critical evaluation of TRPA1-mediated locomotor behavior in zebrafish as a screening tool for novel anti-nociceptive drug discovery. Sci Rep 2019; 9: 2430.
6. Steenbergen, PJ, Bardine, N. Antinociceptive effects of buprenorphine in zebrafish larvae: an alternative for rodent models to study pain and nociception? Appl Anim Behav Sci 2014; 152: 92-99.
7. Lopez-Luna, J, Al-Jubouri, Q, Al-Nuaimy, W, Sneddon, LU. Impact of stress, fear and anxiety on the nociceptive responses of larval zebrafish PLoS ONE 2017; 12(8): e0181010.
8. Lopez-Luna, J, Al-Jubouri, Q, Al-Nuaimy, W, Sneddon, LU. Reduction in activity by noxious chemical stimulation is ameliorated by immersion in analgesic drugs in zebrafish. J Exp Biol 2017; 220: 1451-1458
9. Richendrfer, H, Pelkowski, SD, Colwill, RM, Creton, R. On the edge: pharmacological evidence for anxiety-related behavior in zebrafish larvae. Behav Brain Res 2012; 228: 99–106.

10. Miller, S, Pollack, J, Bradshaw, J, et al. Cardiac responses to hypercapnia in larval zebrafish (*Danio rerio*): the links between CO_2 chemoreception, catecholamines and carbonic anhydrase. J Exp Biol 2014: 217: 3569-3578.

11. Bagatto, B. Ontogeny of cardiovascular control in zebrafish (*Danio rerio*): Effects of developmental environment. Comp Biochem Physiol A 2005; 141: 391 – 400.

12. Coccimiglio, ML, Jonz, MG. Serotonergic neuroepithelial cells of the skin in developing zebrafish: morphology, innervation and oxygen-sensitive properties. J Exp Biol 2012; 215: 3881-3894.

13. Curtright, A, Rosser, M, Goh, S, et al. Modeling Nociception in Zebrafish: A Way Forward for Unbiased Analgesic Discovery. PLoS One 2015; 10(1): e0116766.

14. Deakin, AG, Buckley, J, AlZu'bi, HS, et al. Automated monitoring of behaviour in zebrafish after invasive procedures. Sci Rep 2019; 9(1): 1-13.

15. Ward, JL, McCartney, SP, Chinnadurai, SK, Posner, LP. Development of a minimum-anesthetic-concentration depression model to study the effects of various analgesics in goldfish (*Carassius auratus*). J Zoo Wildlife Med 2012; 43: 214–222.

16. Horsberg, TE, Burka, JF, Tasker, RAR. Actions and pharmacokinetic properties of the α2-adrenergic agents, medetomidine and atipamezole, in rainbow trout (*Oncorhynchus mykiss*). J Vet Anaesthesia 1999; 26: 18-22,

17. Martins, T, Diniz, E, Félix, LM, Antunes, L Evaluation of anaesthetic protocols for laboratory adult zebrafish (*Danio rerio*). PLoS ONE 2018; 13(5): e0197846.

18. Moore, RA, Derry, S, Aldington, D, et al. Amitriptyline for neuropathic pain in adults. Cochrane Database Syst Rev 2015; 2015(7):CD008242.

19. Chervova, LS, Lapshin, DN. Pain sensitivity of fishes and analgesia induced by opioid and nonopioid agents. Proceedings of the Fourth international Iran & Russia Conference on Agriculture and Natural Resources 2004; pp. 1420-1425.

20. Yu, S, Wang, B, Zhang, J, Fang, K. The development of local anesthetics and their applications beyond anesthesia. Int J Clin Exp Med 2019; 12(12): 13203-13220.

21. Westerfield, M. The zebrafish book. A guide for the laboratory use of zebrafish (*Danio rerio*), (1995) 4th ed., Univ. of Oregon Press, Eugene. https://zfin.org/zf_info/zfbook/zfbk.html (accessed 20/03/2023).

22. Readman, GD, Owen, SF, Murrell, JC, Knowles, TG. Do fish perceive anaesthetics as aversive? PLoS One 2013; 8 (9): e73773.

23. Readman, GD, Owen, SF, Knowles, TG, Murrell, JC. Species specific anaesthetics for fish anaesthesia and euthanasia. Sci Rep 2017; 7 (1): 7102.

24. Wong, D, von Keyserlingk, MAG, Richards, JG, Weary, DM. Conditioned place avoidance of zebrafish (Danio rerio) to three chemicals used for euthanasia and anaesthesia. PLoS One 2014; 9 (2): e88030.

25. Collymore, C, Tolwani, A, Lieggi, C, Rasmussen, S. Efficacy and safety of 5 anesthetics in adult zebrafish (*Danio rerio*). J Am Assoc Lab Anim Sci 2014; 53(2): 198-203.

26. Deakin, AG, Spencer, JW, Cossins, AR, et al. Welfare challenges influence the complexity of movement: fractal analysis of behaviour in zebrafish. Fishes 2019; 4(1): 8.

27. Valentim AM, Félix LM, Carvalho L, Diniz E, Antunes LM A New Anaesthetic Protocol for Adult Zebrafish (*Danio rerio*): Propofol Combined with Lidocaine. PLoS ONE 2016; 11(1): e0147747.

28. Sneddon, LU. Clinical Anesthesia and Analgesia in Fish. J Exotic Pet Med 2012; 21(1): 32-43.

29. Nordgreen, J, Tahamtani, FM, Janczak, AM, Horsberg, TE. Behavioural effects of the commonly used fish anaesthetic tricaine methanesulfonate (MS-222) on zebrafish (*Danio rerio*) and its relevance for the acetic acid pain test. PLoS One 2014; 9 (3), e92116.

30. Chance, RJ, Cameron, GA, Fordyce, M, et al. Effects of repeated anaesthesia on gill and general health of Atlantic salmon, *Salmo salar.* J Fish Biol 2018; 93: 1069–1081.

31. Dang, M, Henderson, RE, Garraway, LA, Zon, LI. Long-term drug administration in the adult zebrafish using oral gavage for cancer preclinical studies. Disease Models & Mechanisms 2016; 9: 811-820.

32. Schroeder, P, Lloyd, R, McKimm, R, et al. Anaesthesia of laboratory, aquaculture and ornamental fish: Proceedings of the first LASA-FVS Symposium. Lab Anim. 2021; 55(4): 317-328.

33. Owen, JP, Kelsh, RN. A suitable anaesthetic protocol for metamorphic zebrafish. PLoS One. 2021; 16(3): e0246504

34. Stoskopf, M, Posner, LP. Anesthesia and Restraint of Laboratory Fish. In Fish, R, M, Brown, P, Danneman, A, Karas (Eds.), (2015) *Anesthesia and Analgesia in Laboratory Animals*, 2nd ed. Academic Press, London, UK, pp. 519-534.

35. Ganjoor, M, Salahi-Ardekani M, Nazari S, et al. The Effectiveness of Ketamine as an Anesthetic for Fish (Rainbow Trout – *Oncorhynchus mykiss*). Oceanogr Fish Open Access J 2020; 13(1): 555852.

36. Bruecker, P, Graham, M. The effects of the anesthetic ketamine hydrochloride on oxygen consumption rates and behaviour in the fish *Heros (cichlasoma) citrinellum* (günther, 1864), Comp Biochem Physiol C 1993; 104: 57-59.

37. Al-Hamdani, AH, Ebrahim, SK, Mohammad, FK. Experimental xylazine-ketamine anesthesia in the common carp (*Cyprinus carpio*). J Wildlife Dis 2010; 46(2):596-8.

38. Mylonas, CC, Cardinalettia, G, Sigelaki, I, Polzonetti-Magni, A. Comparative efficacy of clove oil and 2-phenoxyethanol as anesthetics in the aquaculture of European sea bass (*Dicentrarchus labrax*) and gilthead sea bream (*Sparus aurata*) at different temperatures. Aquacult 2005;. 246: 467-481.

39. Musk, GC, Ezzy, BJ, Kenchington, LM, et al. A Comparison of Buffered Tricaine Methanesulfonate (MS-222) and Isoeugenol Anesthesia for Caudal Fin Clipping in Zebrafish (*Danio rerio*). J Am Assoc Lab Anim Sci. 2020: 59(6): 732-736

40. Minter LJ,, Bailey, KM, Harms, CA, et al. The efficacy of alfaxalone for immersion anesthesia in koi carp (*Cyprinus carpio*) Vet Anaesth Analgesia 2014; 41(4): 398-405.

41. Farry, T, Lau, C, Keates, H, et al.. Comparison of two formulations of alfaxalone in laboratory zebra fish (*Danio rerio*) for use in immersion anaesthesia. Vet Anesth Analgesia, 2022; 46(6): 831.e5–831.e6

42. Martins T, Valentim A, Pereira N, Antunes LM. Anaesthetics and analgesics used in adult fish for research: A review. Lab Anim 2019; 53(4):3 25-341. doi:10.1177/0023677218815199

43. Ferreira JM, Jorge S, Félix L, et al. Behavioural Aversion and Cortisol Level Assessment When Adult Zebrafish Are Exposed to Different Anaesthetics. Biology (Basel). 2022; 11(10): 1433. doi: 10.3390/biology11101433.
